# Multiple-Disease Detection and Classification across Cohorts via Microbiome Search

**DOI:** 10.1128/mSystems.00150-20

**Published:** 2020-03-17

**Authors:** Xiaoquan Su, Gongchao Jing, Zheng Sun, Lu Liu, Zhenjiang Xu, Daniel McDonald, Zengbin Wang, Honglei Wang, Antonio Gonzalez, Yufeng Zhang, Shi Huang, Gavin Huttley, Rob Knight, Jian Xu

**Affiliations:** a Single-Cell Center, Qingdao Institute of BioEnergy and Bioprocess Technology, Chinese Academy of Sciences, Qingdao, Shandong, China; b Department of Pediatrics and Center for Microbiome Innovation, University of California at San Diego, San Diego, California, USA; c Departments of Computer Science and Engineering and Bioengineering, University of California at San Diego, San Diego, USA; d Research School of Biology, ANU College of Science, The Australian National University, Canberra, Australia; e University of Chinese Academy of Sciences, Beijing, China; University of Massachusetts Medical School

**Keywords:** microbiome, search, disease detection and classification

## Abstract

Microbiome-based disease classification depends on well-validated disease-specific models or *a priori* organismal markers. These are lacking for many diseases. Here, we present an alternative, search-based strategy for disease detection and classification, which detects diseased samples via their outlier novelty versus a database of samples from healthy subjects and then compares these to databases of samples from patients. Our strategy’s precision, sensitivity, and speed outperform model-based approaches. In addition, it is more robust to platform heterogeneity and to contamination in 16S rRNA gene amplicon data sets. This search-based strategy shows promise as an important first step in microbiome big-data-based diagnosis.

**IMPORTANCE** Here, we present a search-based strategy for disease detection and classification, which detects diseased samples via their outlier novelty versus a database of samples from healthy subjects and then compares them to databases of samples from patients. This approach enables the identification of microbiome states associated with disease even in the presence of different cohorts, multiple sequencing platforms, or significant contamination.

## Abstract (precis)

Here, we present a search-based strategy for disease detection and classification, which detects diseased samples via their outlier novelty versus a database of samples from healthy subjects and then compares them to databases of samples from patients. This approach enables the identification of microbiome states associated with disease even in the presence of different cohorts, multiple sequencing platforms, or significant contamination.

## INTRODUCTION

**FIG 1:**
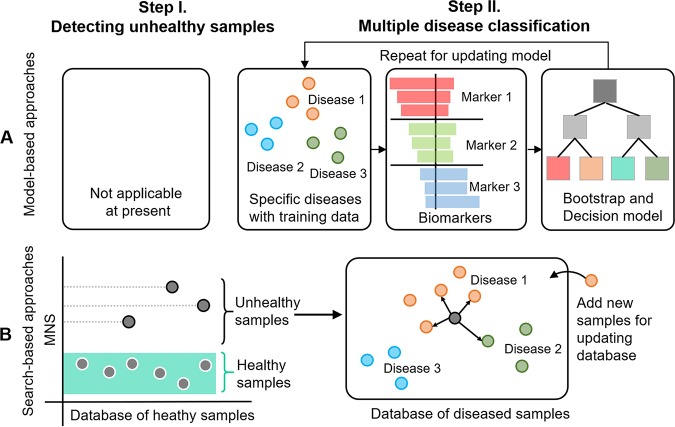
Comparison of model-based and search-based diagnosis. (A) The model-based approach starts by defining specified targeted diseases with training samples and then selects biomarkers for modeling. The whole procedure is repeated for updating the model upon availability of new training samples. (B) The search-based approach starts by detecting disease samples based on their outlier MNS compared to a comprehensive healthy-sample database, without making any *a priori* hypothesis about any diseases, and then performs multiple-disease classification for the unhealthy samples based on their nearest compositional matches in a database of samples from diseased subjects. The two databases are updated when additional reference microbiomes are available.

Microbiome-wide association studies have found diagnostic (and prognostic) applications in many diseases (1). Current strategies for such diagnosis typically build computational models by identifying organismal or gene-based biomarkers from specifically selected cohorts with validated samples from patients and their healthy controls via machine learning methods. These models are then applied to the query to derive a numeric index of disease status, severity, or risk (Fig. 1A). However, such models generally require an *a priori* assumption (and samples) of a particular disease and its corresponding control samples from healthy subjects. In addition, extending a model to other studies, even of the same disease, can be challenging, since selection of organismal biomarkers generally requires careful consideration of the effects of a plethora of factors, including host metadata (e.g., age, disease stage, etc. [2]) and sequencing technologies. Moreover, organismal biomarkers can be associated with multiple diseases, which can cause misclassification (3). Because efforts to systematically evaluate and curate disease-specific statistical models have only just begun (4), their availability for use in diagnosis is limited.

Here, we present an alternative, search-based strategy for disease detection and classification. Using a database of samples from healthy subjects as a reference distribution we employ an outlier detection strategy to identify disease status. Then, disease classification is achieved by subsequent comparison to databases of samples from patients. Using standard performance measures, the precision, sensitivity, and speed of our search-based method outperform the widely used model-based approaches of random forest (RF), support vector machine (SVM), and eXtreme Gradient Boosting (XGBoost). In addition, we demonstrate that our approach is more robust to platform heterogeneity and contamination in 16S rRNA gene amplicon data sets.

## RESULTS

### Search-based disease detection and multiple classification with a two-step process.

We recently developed Microbiome Search Engine (MSE), which rapidly and precisely identifies matches to a query sample from hundreds of thousands of known microbiomes based on their phylogeny-based compositional similarity (5). We hypothesized that such a search capability, which is at the whole-microbiome level, could be exploited to address the aforementioned limitations of model-based approaches (Fig. 1A). To test this hypothesis, we proposed a search-based strategy for microbiome-based diagnosis using MSE in two steps (Fig. 1B). (i) We assign disease status to samples identified as outliers relative to a large, comprehensive database of samples from healthy subjects (i.e., a baseline database); (ii) candidate disease(s) are then selected via multiple classification of diseases performed across cohorts via similarity to a database of samples from diseased subjects.

**Step I: The disease status of a sample is determined via an outlier microbiome novelty score (MNS) against the baseline database.** Taxonomic profiles are available for tens of thousands of human-associated microbiomes on the operating taxonomy unit (OTU) level that have been generated by a large number of studies via sequencing 16S rRNA gene amplicons. Because the diversity of human microbiomes from healthy subjects in 16S studies is nearly saturated as defined by the MNS (5) (see Fig. S1 in the supplemental material), we hypothesized that healthy human microbiome data could be used as a baseline to predict disease status, because samples from individuals with disease were expected to exhibit extreme MNS values (Fig. 1B). To test this hypothesis, we first established a fecal baseline database which consisted of all human fecal samples from healthy subjects from the Qiita database (http://qiita.ucsd.edu; 6) (*n *= 15,704 from 56 studies and 94 countries/regions; both adults and children; Table S1). We constructed a test data set, Data Set Gut, which included fecal microbiomes assessed by 16S rRNA gene amplicon profiling (*n *= 3,113 from 9 studies, excluded from the baseline database; see Materials and Methods; Table S2). Specifically, Data Set Gut contained samples derived from individuals without disease (healthy controls) and from individuals diagnosed with a disease, either inflammatory bowel disease (IBD), human immunodeficiency virus (HIV), colorectal cancer (CRC), or enteric diarrheal disease (EDD).

10.1128/mSystems.00150-20.1 FIG S1 The temporal accumulation of total microbiomes and novel microbiomes for human gut baseline (i.e., healthy). The novel samples are identified based on the threshold of mean MNS in 2010. The novel sample rate was developed in a descending trend and kept in a quite low level that was less than 5%. Source data are provided as Data Set S1. Download FIG S1, TIF file, 0.5 MB. Copyright © 2020 Su et al. 2020 Su et al. This content is distributed under the terms of the Creative Commons Attribution 4.0 International license.

10.1128/mSystems.00150-20.4 TABLE S1 The fecal samples of the baseline database for search-based diagnosis. Download Table S1, DOCX file, 0.1 MB. Copyright © 2020 Su et al. 2020 Su et al. This content is distributed under the terms of the Creative Commons Attribution 4.0 International license.

10.1128/mSystems.00150-20.5 TABLE S2 Data Set Gut for evaluation of search-based diagnosis. Download Table S2, DOCX file, 0.04 MB. Copyright © 2020 Su et al. 2020 Su et al. This content is distributed under the terms of the Creative Commons Attribution 4.0 International license.

**FIG 2:**
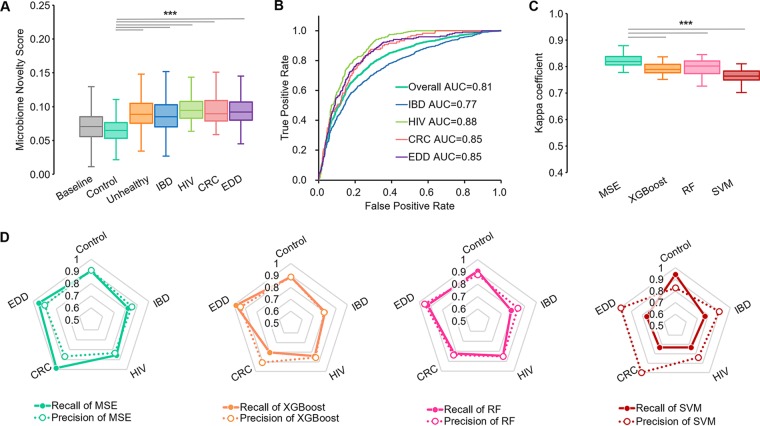
Microbiome search-based disease status detection and classification. (A) MNS of gut samples from patients (IBD, HIV, CRC, EDD, or their pools) are significantly different from those of samples from healthy subjects. (B) Receiver operating characteristics (ROCs) of MSE-based disease prediction. (C) Comparison of MSE-based and model-based (XGBoost, RF, and SVM) methods for performance via Kappa coefficients. (D) Recall and precision of MSE versus models for each cohort. Each vertex of the pentagon represents a recall/precision value on a specific disease, and thus, collapse on any vertex reflects shortcoming of the method in detecting the corresponding disease. For boxplots in A and C, central lines represent the medians, the bounds of the box represent the quartiles, and error bars represent the local maximum and local minimum values. ***, *P* < 0.01. Source data are provided as Data Set S1.

Each of the 3,113 samples in Data Set Gut was searched against the baseline database to calculate its MNS, quantifying the degree of structural dissimilarity between a query microbiome and those in the healthy baseline database (5) (see Materials and Methods). The MNS of the healthy control samples in Data Set Gut were significantly lower than those from individuals with disease (Wilcoxon rank-sum test *P* value < 0.01, corrected by removing longitudinal replicates; Fig. 2A). Such extreme taxonomic compositions relative to the baseline data can be exploited for the detection of samples with disease. In fact, this MNS-based detection reached an area under the curve (AUC) of 0.81 (a maximum statistical F1-score of 0.78 was reached when the MNS was set to 0.072; recall = 0.80; precision = 0.75) for Data Set Gut (Fig. 2B) in the absence of any preexisting knowledge of disease.

**FIG 3:**
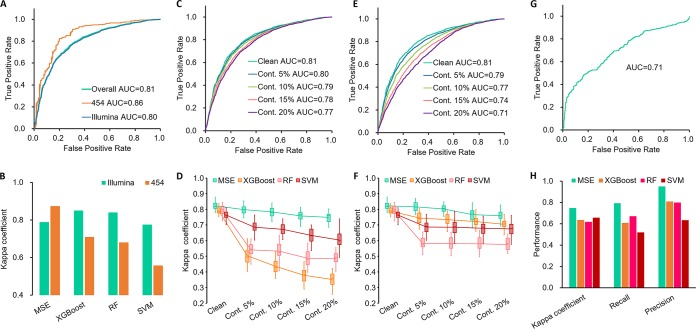
Robustness of MSE to sequencing platform change and DNA contamination. Gut microbiome samples were used as the example. (A) ROCs of MNS-based disease status prediction under two sequencing platforms. (B) Difference in Kappa coefficient (*k*) of disease classification under two sequencing platforms. ROCs of MNS (C) and variation of *k* for multiple-disease classification (D) with reagent blank microbiome contaminations. ROCs of MNS (E) and variation of *k* of multiple disease classification (F) with indoor environmental contaminations. (G) ROC of MNS-based disease detection by independent IBD cohorts and (H) disease classification for cross-cohort data sets by MSE and by model-based approaches. For boxplots in D and F, central lines represent the medians, the bounds of the box represent the quartiles, and error bars represent the local maximum and local minimum values. Source data are provided as Data Set S1.

**FIG 4:**
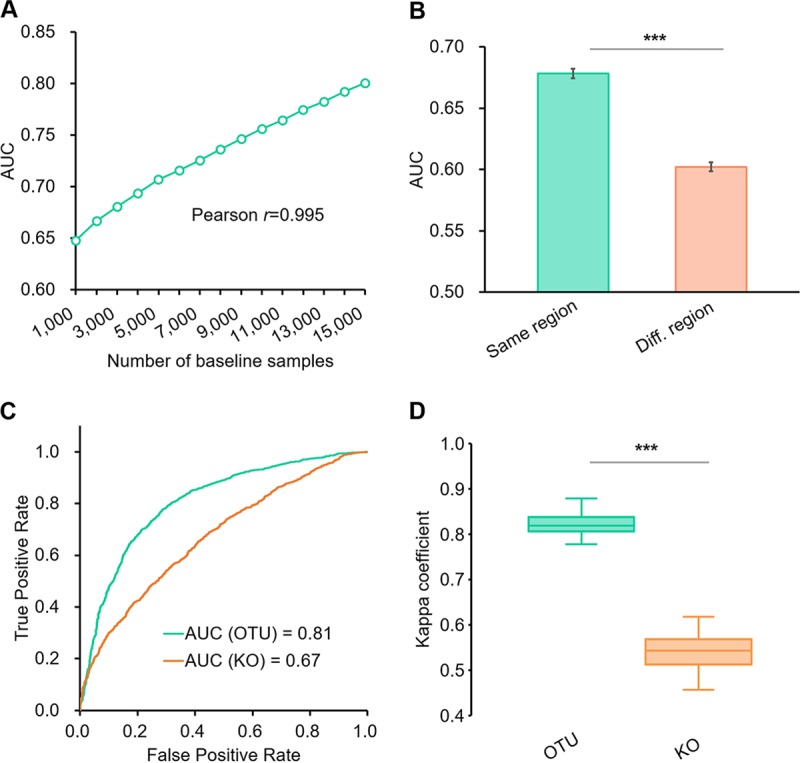
Microbiome data features that influence the performance of search-based disease detection with gut samples. (A) The AUC of MNS-based disease detection is affected by the number of baseline samples. (B) The AUC of MNS-based disease detection is affected by the geographic regions of baseline samples. Error bars represent the standard deviation. (C) OTU-based search has higher AUC than KO-based search in detecting disease samples by MNS. (D) Kappa coefficients of OTU-based multiple classification are significantly higher than those of the KO-based method. Central lines represent the medians, the bounds of the box represent the quartiles, and error bars represent the local maximum and local minimum values. ***, *P* < 0.01. Source data are provided as Data Set S1.

**FIG 5:**
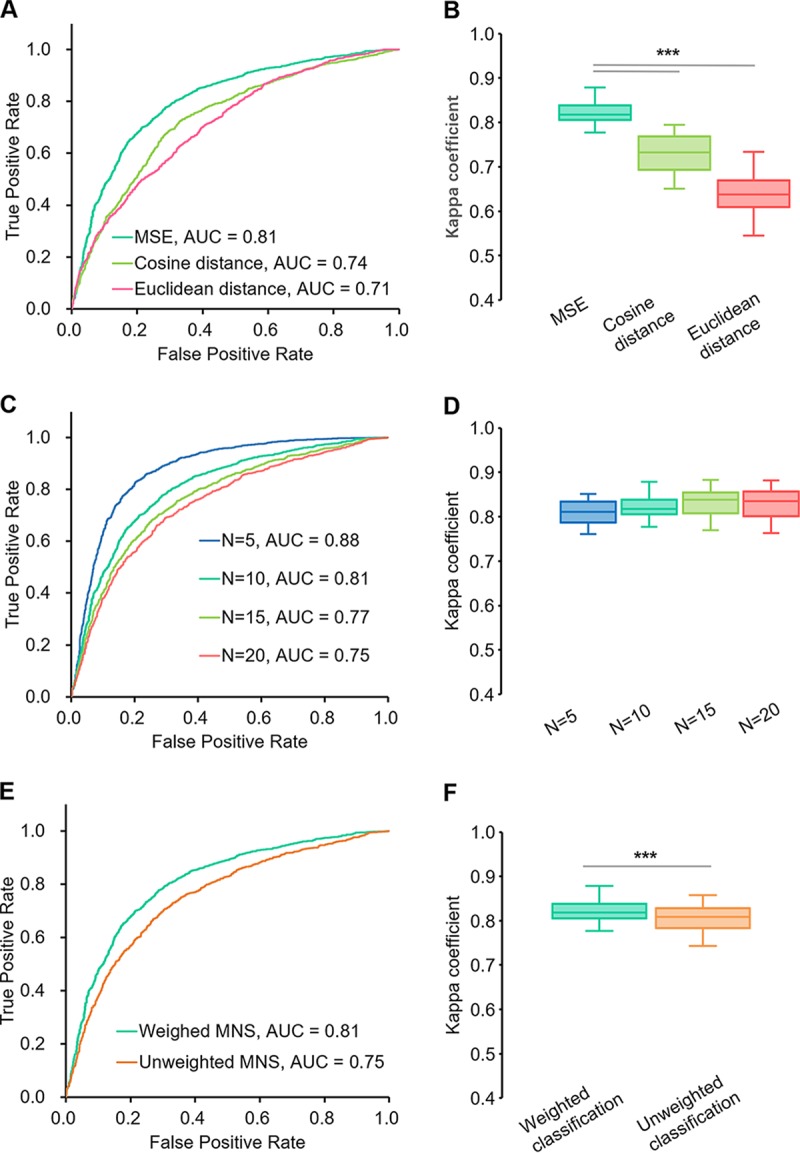
Search parameters that influence the performance of MSE-based disease detection and classification. (A) ROCs of MNS-based disease status detection in MSE step I based on different distance metrics. (B) Kappa coefficients (*k*) of multiple-disease classification in MSE step II based on distinct distance metrics. (C) ROCs of MNS-based disease status detection in MSE step I based on different numbers of matches. (D) *k* of multiple-disease classification in MSE step II based on distinct numbers of matches. (E) ROCs of MNS-based disease status detection based on weighted or unweighted MNS. (F) *k* of multiple-disease classification based on weighted or unweighted classification. For boxplots in B, D, and F, central lines represent the medians, the bounds of the box represent the quartiles, and error bars represent the local maximum and local minimum values. ***, *P* < 0.01. Source data are provided as Data Set S1.

10.1128/mSystems.00150-20.8 DATA SET S1 The source data underlying Fig. 2A to D, 3A to H, 4A to D, 5A to F and Fig. S1, S2, and S3. Download Data Set S1, XLSX file, 1.6 MB. Copyright © 2020 Su et al. 2020 Su et al. This content is distributed under the terms of the Creative Commons Attribution 4.0 International license.

**Step II: Unhealthy samples detected in step I are further searched against a curated database of microbiomes from diseased subjects by MSE for multiple disease classification.** After the identification of disease status in step I, the goal is to pinpoint the specific underlying disease (Fig. 1B). To test the accuracy of MSE-based diagnosis across cohorts, Data Set Gut was analyzed for 10-fold cross-validation, repeated 30 times, while models were constructed using XGBoost, RF, and SVM in parallel (see Materials and Methods). Single samples were chosen from host individuals that had been sampled multiple times during the cross-validation to avoid statistical bias (see Materials and Methods). We then used the Kappa coefficient (*k*) to compare the overall performance of MSE with these three model-based approaches on multicohort classification which measures the rates of both correctly classified queries and misclassified ones. (The AUC performance measure is for binary discrimination and is not well suited to more than two categories.) Among the five cohorts (IBD, HIV, CRC, EDD, and control) of Data Set Gut, MSE achieved a *k* of 0.85 ± 0.03, indicating a striking agreement with the experimental design (Fig. 2C). The distribution of *k* from MSE in the 30 repetitions was significantly higher than that of all the model-based approaches (paired Wilcoxon rank-sum test *P* value < 0.01; refer to Table 1 for statistical details; Fig. 2C). Furthermore, MSE-based classification features less weakness of recall and precision for each of the four diseases; in contrast, the model-based metrics suffer from certain obvious biased performance, such as lower recall of identifying CRC for XGBoost, IBD for RF, and EDD and CRC for SVM (Fig. 2D).

**TABLE 1:** Statistical details of the Kappa coefficients for each method

Method^*a*^	Repetition (time)	Mean	SD	95% CI^*b*^	*P* value to MSE^*c*^
MSE	30	0.822	0.025	0.813–0.831	
XGBoost	30	0.789	0.026	0.779–0.798	2.762e-06
RF	30	0.792	0.036	0.778–0.805	3.128e-04
SVM	30	0.763	0.028	0.753–0.774	1.863e-09
MSE (KO)	30	0.543	0.050	0.524–0.561	1.863e-09
MSE (cosine distance)	30	0.729	0.041	0.713–0.744	1.863e-09
MSE (Euclidean distance)	30	0.636	0.049	0.618–0.654	1.863e-09
MSE (*N* = 5)	30	0.810	0.026	0.800–0.819	0.184
MSE (*N* = 15)	30	0.832	0.029	0.822–0.843	0.191
MSE (*N* = 20)	30	0.832	0.032	0.820–0.844	0.262
MSE (Unweighted)	30	0.806	0.029	0.795–0.817	4.408E-05

a *N*, number of search matches.

b CI, confidence interval.

c *P* values were calculated using the Paired Wilcoxon test.

### Robustness of MSE to technical data variation and to contamination.

**Robustness to sequencing platform heterogeneity.** Heterogeneity in sequencing platform is frequently encountered in cumulative disease-specific microbiome data sets. This has become a hurdle for cross-cohort application of microbial disease markers (7). We tested the robustness of MSE to such technical data variation. In step I of MSE, the Roche 454 and Illumina sequences in Data Set Gut carry an AUC of 0.86 and 0.80, respectively, which are both close to the overall AUC of 0.81 (Fig. 3A). In step II, the *k* of >0.8 by leave-one-out cross-validation (LOOCV) also supports excellent performance of MSE in the simultaneous presence of the two sequencing platforms (0.79 for Illumina and 0.87 for 454; Fig. 3B). In contrast, accuracy of the model-based diagnosis is heavily dependent on sequencing platform variation (8), as suggested by the preference for Illumina samples over 454 samples (Fig. 3B). Thus, MSE was less affected by the change in sequencing platform than the model-based approaches. This is a key advantage in the reuse of data to avoid the per-study bias (7) due to the sequencing-platform variation for 16S amplicon-based data sets.

**Robustness to sequence contamination.** Another factor that greatly affects the accuracy of model-based disease classification is contamination by DNA from the experimental workflow or from the environment. To probe the robustness of MSE to this problem, randomly selected OTUs from two different sources of contamination—reagent blank microbiomes and indoor environment microbiomes—were mixed into samples in testing Data Set Gut (see Materials and Methods). These represent the most likely source of contamination for human microbiome samples in the context of disease diagnosis. The rate of contamination was set to 5%, 10%, 15%, and 20%. Even at a high contamination rate of 20%, MSE still offers a reasonable performance, in both disease status detection (i.e., step I; AUC = 0.77 with reagent blank contaminations in Fig. 3C; AUC = 0.71 with indoor contaminations in Fig. 3E) and disease classification (i.e., step II; *k *= 0.75 ± 0.03 in Fig. 3D; *k *= 0.76 ± 0.03 in Fig. 3F). Furthermore, in step II the robustness to reagent blank contaminations of MSE was 3.2-fold higher than that of all three model-based methods (average Δ*k* with 20% OTU contamination versus with 0% OTU contamination: 9.0% versus 38.4%; Fig. 3D), whereas robustness to indoor environment contamination was 2.1-fold higher than that of model-based methods (average Δ*k*: 7.6% versus 16.5%; Fig. 3F). Specifically, when 20% blank contaminated OTUs were present in a given query sample, MSE still featured recall of 83.4% and 76.6% in detecting HIV and IBD, respectively. In contrast, the three model-based methods showed a reduction in recall to 32.0% and 37.5%, respectively, on average. Thus, MSE was much more robust to DNA contamination than model-based methods.

**Stability across unrelated studies.** To test the stability of MSE performance across unrelated studies, fecal microbiome samples from a second, independent IBD cohort were analyzed (9) (*n =* 375 and 393 for samples from diseased and healthy subjects, respectively, which were not included in the baseline database and Data Set Gut). In step I, the MNS of patient samples were significantly higher than those of controls (Wilcoxon rank-sum test *P* value < 0.01), based on which they were detected with AUC = 0.71 (Fig. 3G) without *a priori* knowledge. Searching against Data Set Gut, IBD status was identified with *k *= 0.75 (recall = 0.79 and precision = 0.95). This compared favorably to an average *k* of 0.52 (recall = 0.47 and precision = 0.67) for model-based approaches using Data Set Gut as training (Fig. 3H). Therefore, MSE’s performance was stable across independent studies of IBD.

### Data features that influence disease detection and classification.

**Size of the baseline database.** As MNS is derived in reference to samples from healthy subjects, we evaluated the effect of baseline database size on the accuracy of MSE step I. The size of the fecal baseline database was rarefied from *n *= 1,000 to 15,000 with step 1,000. This was repeated 10 times. Step I was performed to detect samples with disease in Data Set Gut using the rarefied baselines. There was a strong positive correlation between AUC and baseline database size (Pearson *r *= 0.995; Fig. 4A). For instance, when the baseline database size was *n* = 1,000 healthy microbiomes, the AUC was 0.65, in contrast to an AUC of 0.81 for *n* = 15,704. Therefore, the number of healthy control samples is a crucial determinant of MSE performance. This result indicates that MSE performance can still be improved with the accumulation of more microbiomes from healthy individuals (Fig. 4A). Given that the number of shotgun-sequenced samples from healthy subjects remains a tiny fraction of those 16S rRNA genes sequenced, the results also underscore the key advantage of 16S data sets for search-based diagnosis.

**The accuracy of MSE-based disease detection differed between geographic regions**. Since the diversity of the human microbiome is strongly associated with the geographical region (10), we assessed whether geographical sampling coverage in the baseline database affected MSE step I performance. The human hosts in Data Set Gut primarily reside in the United States (*n *= 1,832), Sweden (*n *= 695), the United Kingdom (*n *= 185), and Australia (*n *= 26), with the remaining being from 61 other countries/regions (*n *= 375). We split the baseline database into two subsets, those from the same regions as Data Set Gut (*n *= 12,892) and those from regions different from Data Set Gut (*n *= 2,812). Each of the two subsets were then separately used as a baseline database for detecting the unhealthy microbiome in Data Set Gut. To avoid the bias of uneven sample amount, the subset from the original regions was rarefied to *n *= 2,000 samples (with 10 replicates) to avoid the random bias. The baseline from the same regions as the test set yielded higher precision (AUC = 0.68 ± 0.004) than that from different regions (AUC = 0.60 ± 0.004) in detecting diseased samples (Wilcoxon rank-sum test *P* value < 0.01; Fig. 4B). Therefore, MSE performance is affected by the representation of the query samples’ geographic region in the baseline database.

**Taxonomic structure provided higher accuracy in MSE diagnosis than functional profiles.** Here, we also employed the KEGG Orthology (KO)-based search (see Materials and Methods) using PICRUSt (11) inferred functional profiles of 16S data for step I and step II instead of the OTU. In step I, the AUC of 0.67 from the KO-based search for disease detection on Data Set Gut was markedly lower than that from the OTU-based search (AUC = 0.81; Fig. 4C). In step II for disease classification, the *k* coefficients of the KO-based search were significantly lower than that from the OTU-based approach on the test data set (paired Wilcoxon rank-sum test *P* value < 0.01; refer to Table 1 for statistical details; Fig. 4D). Thus, OTU-based MSE performed better than function-based MSE in our tests. There are a number of possible explanations for this observation. First, in PICRUSt, the KO profiles are derived by the products of (i) the abundance of contributed OTUs, (ii) the 16S rRNA gene copy number of the contributed OTUs, and (iii) the KO weight of contributed OTUs. Second, the KO profiles do not contain additional information to measure the relationships among compositional features such as the phylogeny of OTUs, which provides higher accuracy in computing the similarity (see Materials and Methods for details). Third, in machine learning-based classification of biologically meaningful categories, PICRUSt-predicted KO profiles do not necessarily offer improvement over microbial composition data alone (and might actually offer worse results if the aim is to obtain biomarkers of physiological or ecological states [12]). As a next step, it will be intriguing to compare the performance of OTU-based MSE versus searches via functional assignment of shotgun reads once more shotgun metagenomic data sets are available.

### Search parameters that influence performance of MSE-based diagnosis.

We compared the influence of computing sample similarity by a phylogeny-based algorithm (refer to Materials and Methods for details) to that of cosine distance and Euclidean distance on the performance of step I and step II. In step I, MSE achieved an AUC of 0.81 for disease detection, which is higher than results based on cosine distance (AUC = 0.74) or Euclidean distance (AUC = 0.71; Fig. 5A). In step II, the disease classification using MSE achieved a mean *k* of 0.82, significantly higher than that achieved using cosine distance (*k *= 0.73) or Euclidean distance (*k *= 0.64; Fig. 5B; Table 1). Thus, phylogeny-based similarity among microbiome samples can provide higher accuracy in disease detection and classification.

The link between MSE performance and the number of search matches (*N*) was evaluated for MNS calculation (*N* in Equation 4 below; see Materials and Methods) in step I and for disease classification (*N* in Equation 8 below) in step II. *N* was set to 5, 10, 15, and 20. For step I, increasing *N* skewed the detection results and decreased the AUC (Fig. 5C). For step II, *k* remained stable with the change of *N* (Table 1; Fig. 5D). Therefore, *N *= 10 was chosen as a balanced parameter for the two-step diagnosis in MSE.

We probed the influence of the weights for MNS and classification on the diagnosis. For both MNS (Equation 4 below; see Materials and Methods) and disease classification (Equation 8 below), we applied the ranks of matches as weights. In both step I and step II, weighted equations performed better than unweighted ones as follows: AUC of 0.81 for weighted MNS versus 0.75 for unweighted MNS in step I (Fig. 5E) and mean *k* coefficients of 0.82 for weighted classification versus 0.81 for unweighted classification in step II (paired Wilcoxon rank-sum test *P* value < 0.01; see Table 1 for statistics; Fig. 5F). Therefore, weights that favor higher-ranked matches improve the performance of MSE-based predictions.

### Rapid microbiome classification by MSE.

By developing a highly efficient indexing strategy that identifies matching candidates, MSE features rapid search, i.e., within 0.3 sec for a complete two-step diagnosis against a 15,000-sample database. In MSE, update or expansion of databases is faster and easier than in model-based approaches. The latter require that the statistical model separating cases from controls be retrained from scratch each time new samples are added (Fig. S2). Furthermore, for MSE, the search speed is quite stable and much less sensitive to database size increase than exhaustive search (see Materials and Methods), and optimizations, including data reencoding and memory allocation already enable MSE to handle large-scale data sets at the million-sample level (Fig. S3). Thus, with the rapid accumulation of microbiome data sets globally, the performance gap in speed is expected to widen as more samples are added.

10.1128/mSystems.00150-20.2 FIG S2 Running time of training with different numbers of training samples and classifying 100 testing samples. For model-based machine learning methods, the total running time includes both training time and testing time. For MSE, the total running time was for a complete two-step diagnosis with database construction and search. All tests were performed on a single rack server node with Quad Intel Xeon E7-4820 CPUs. The *y* axis is in log scale. Source data are provided as Data Set S1. Download FIG S2, TIF file, 0.2 MB. Copyright © 2020 Su et al. 2020 Su et al. This content is distributed under the terms of the Creative Commons Attribution 4.0 International license.

10.1128/mSystems.00150-20.3 FIG S3 The increase of mean search time for a single search by MSE and exhaustive search with the increase of database size. All tests were performed on a single rack server node with Quad Intel Xeon E7-4820 CPUs. Source data are provided as Data Set S1. Download FIG S3, TIF file, 0.3 MB. Copyright © 2020 Su et al. 2020 Su et al. This content is distributed under the terms of the Creative Commons Attribution 4.0 International license.

## DISCUSSION

Here, via MSE, we show that microbiome big data assembled from the many but diverse past microbiome-sequencing studies can serve as a basis for microbiome-based disease diagnosis. We employ a two-step process in which a query is evaluated first against a baseline database of microbiomes from healthy individuals, followed by comparison against a database of disease-specific samples. The first step constitutes an outlier detection strategy. The second uses a search-based k-nearest-neighbor (KNN)-like classification strategy. The resulting predictions provide direction and hypotheses for clinical decision-making. The two-stage approach provides considerable computing performance advantages for continuous updating of the microbiome databases to incorporate new studies compared to alternate model-based approaches. Moreover, MSE does not require curation of disease-specific models or biomarkers. MSE also provides a new approach to disease prediagnosis, i.e., deciding whether the microbiome is indicative of a disease status.

In light of the general shift of microbiome-sequencing focus from healthy to diseased hosts (13–15), the findings here advocate for increased geographic sequencing of additional baseline samples. At present, in the healthy gut microbiome database, over 80% of the samples are from the United States, the United Kingdom, and other European countries, while other populations are poorly represented. As the accuracy of MSE prediction is dependent on the number of microbiomes, the underrepresented populations should be prioritized for healthy microbiome sampling. However, healthy microbiomes from distant populations are also of value; for example, for IBD diagnosis in U.S. patients, using healthy non-U.S. samples instead of healthy U.S. samples as a baseline generates an AUC of 0.68 (reduced from 0.79), although even this lower AUC is still meaningful. Thus, a coordinated effort with global sampling coverage is warranted.

On the other hand, despite its performance advantages when tested on human gut microbiomes for several disease cohorts, this type of search-based classification strategy will not offer better performance than other machine learning methods under all circumstances (in accordance with the “no free lunch” theorem in machine learning). For example, for classification of human body sites from microbiome data, the accuracy of MSE is equivalent to or lower than several machine learning approaches we tested (Table S4). Therefore, the potential, as well as limitations, of MSE in predicting the origin, state, and function (or in general, any of the metadata) of a microbiome sample need to be explored in future studies to develop general guidelines for applicability. Nevertheless, we expect that search via MSE will become an important first step in any microbiome-based diagnosis, just as a BLAST search is in sequence-based diagnosis today.

## MATERIALS AND METHODS

### Data sets for testing MSE performance.

Data Set Gut (Table S2) consists of 3,113 fecal gut microbiome samples collected from eight studies, among which, 993 samples were clinically identified as inflammatory bowel disease (IBD), 120 as colorectal cancer (CRC), 360 as HIV, 222 as enteric diarrheal disease (EDD), and 1,418 samples as healthy controls. These 16S rRNA gene amplicons were from various regions (e.g., V1-V2, V1-V3, V3-V4, V4, and V3-V5), and sequences were produced using a Roche 454, Illumina MiSeq, or Illumina HiSeq system.

### Processing of 16S rRNA gene amplicon data for MSE.

All microbiome samples were processed from their published sequence data using Parallel-META 3 version 3.4.3 (16) with Greengenes 13-8 (17) on the OTU similarity level of 97%. Variation of 16S rRNA gene copy number was normalized based on the Integrated Microbial Genomes (IMG) database (18) to maximally reduce the bias of comparison with samples from different platforms and studies. The functional profiles were predicted with the PICRUSt (11) algorithm and annotated with KEGG Orthology. We set a minimum sequence number of 500 and a minimum 16S rRNA gene mapping rate of 80% for each sample to ensure high quality. Output of this procedure then serves as the input to MSE.

### Brief overview of MSE.

MSE aims to rapidly identify the structurally “similar” microbiomes of a given query microbiome from a large-scale depository of known microbiomes (5). The search module of MSE consists of a series of algorithms that perform two phases. In the “database construction” phase, MSE builds the search index for all database samples by partitioning their relative abundance of operational taxonomy units (OTUs) into index keys for fast fetch. Next, the “database search” phase is a two-tier process as follows: (i) with a given query sample, MSE performs index fetching by calculating its index keys and then selects a constant number (e.g., 200) of “candidate matches” that have the shortest distances to the query on index keys; (ii) MSE evaluates the phylogeny-based microbiome similarity (defined below) by a pairwise comparison between the query and each of the “candidate matches” via the Meta-Storms algorithm (19) on the OTU level, so as to precisely identify the top matches.

Defining *T_similarity_* as the time taken to calculate the phylogeny-based similarity between two microbiomes in a database with *N* samples, the time consumed for a single exhaustive search is

(1) $$T_{\mathrm{exhaustive}}=N\times T_{\mathrm{similarity}}$$

In comparison, defining the dynamic index-fetching time as *T_indexing_*, the time taken for a single dynamic index-based search by MSE is

(2) $$T_{\mathrm{MSE}}=T_{\mathrm{indexing}}+200\times T_{\mathrm{similarity}}$$

Actually, *T_indexing_* « 200 × *T_similarity_*, even for searches in a database with one million samples. Therefore, the search time can be theoretically approximated as

(3) $$T_{\mathrm{MSE}}\approx 200\times T_{\mathrm{similarity}}$$

We found that for a single search in the database with 10,000 samples, the exhaustive search took 1.15 s, and MSE used 0.14 s (Fig. S3). When the database size increased to 1,000,000 (100 times bigger), the time of exhaustive search exhibited a linear increase with database size of 99.06 s. MSE finished in 0.29 s (with 98.84% consistency in search result to the exhaustive search; Fig. S3), which is 340 times faster than exhaustive search. Therefore, the querying speed of MSE is much less sensitive to database size than exhaustive search.

### Calculation of microbiome novelty score.

The microbiome novelty score (MNS) is proposed to evaluate the compositional uniqueness of a microbiome sample compared to a reference microbiome database (5). With a given query sample *q* and its top *N* matches, for its match *i*, whose microbiome similarity is *S_i_* (see equation 5), the MNS(*q*) was calculated from the top *N* ordered matches (we used *N* = 10)

(4) $$\mathrm{MNS}=1-\frac{\sum_{i=1}^{N}\left(S_{i}\times \left(N-i+1\right)\right)}{\sum_{i=1}^{N}\left(N-i+1\right)}$$

For each microbiome sample, its MNS was derived by searching it against all samples in the reference database. Thus, a higher MNS means lower similarity to those microbiomes in the database, suggesting higher novelty and uniqueness.

### Defining the microbiome similarity score.

We used a microbiome similarity measure that has a strong correlation with the UniFrac metric (Spearman *r *= 0.915 [20]) and is optimized for large-scale parallel computing. The phylogeny-based similarity of two microbiomes (20) assumes a phylogenetic tree representing the relationships between the union of sequences from the samples. To compute a similarity between two samples, we first define the node weights for an internal node of the tree as

(5) $$\omega_{j}=\left|\omega_{0}-\omega_{1}\right|\left(1-\ell_{j}\right)$$

where $\ell_{j}$ is the branch length leading to node *j*. Thus, for node *i*, with descendants *j* and *k*, the node weights are

(6) $$\psi \left(i\right)=\{\omega_{j},\omega_{k}\}$$

If node *i* is a terminating edge, then *Ψ* is the unordered set of abundance of OTU *i* in the two samples. The similarity, *S*, of the two samples is then

(7) $$S=\overset{n}{\underset{i=1}{\sum }}\min\psi \left(i\right)$$

where *n* is the number of nodes on the tree.

### Search-based classification of multiple disease status.

The disease status of a new microbiome is predicted based on the metadata of its top *N* matches (we used *N *= 10). For a status metadata, G = {g_1_, g_2_, …., g_m_} for healthy status (e.g., in Data Set Gut, *m *= 5, g_1_ = IBD, g_2_ = HIV, g_3_ = CRC, g_4_ = EDD, and g_5_ = control), and with a given query sample *q* and its top *N* matches, the microbiome similarity to match *i* is *S_i_*, and then the score of query sample *q* for status g_k_ is

(8) $$\mathrm{Score}(q\in g_{k})=\frac{\sum_{i\in g_{k}}S_{i}(N-i+1)}{\sum_{j=1}^{N}S_{j}(N-j+1)}$$

Thus, the final classification is determined by the status with the maximum score across all cohorts.

### Model-based classification of multiple disease status.

We constructed the machine learning model for multiple disease diagnosis using three metrics of random forest (RF), support vector machine (SVM), and eXtreme Gradient Boosting (XGBoost). All models were trained and verified using the taxonomic relative abundances on OTU level after 16S rRNA gene normalization. The RF model was trained by the randomForest package in R; the SVM model was trained by the svm function in R; the XGBoost model was trained by the xgboost package in R. The machine learning training processes were performed with optimized parameters for multicategory classification for microbiome data using the strategy introduced by Statnikov et al. (21) (Table 2).

**TABLE 2:** Optimized parameters for machine learning-based multiple classification

Method	Parameter^*a*^	Value
RF	ntree	2,000
RF	mtry	2 × $\sqrt{\text{\# of OTU}}$
SVM	Kernel	Polynomial
SVM	Degree	3
XGBoost	Objective	multi:softmax
XGBoost	nrounds	20

a ntree, number of trees; mtry, number of variables sampled at each split; nrounds, maximum number of iteration times.

### Calculation of the Kappa coefficient.

The area under the receiver operating characteristic curve (AUC) is usually used to measure the performance of a model for discrimination between binary states (e.g., a specific disease versus healthy control). Because AUC is not available with a status of ≥3, we used the Kappa coefficient (*k*) to evaluate the performance of MSE in multistate classification. The Kappa coefficient quantifies the consistency of the sample distribution in multiple cohorts between the classification results of MSE and the known distribution. *k* is always between 0 and 1, and the following intervals provide guidance for interpretation: 0.00 to 0.20, slight consistency; 0.21 to 0.40, fair consistency; 0.41 to 0.60, moderate consistency; 0.61 to 0.80, substantial consistency; and 0.81 to 1.00, almost perfect consistency. The recall (also referred to as sensitivity; true positive/[true positive + false negative]) and precision (true positive/[true positive + false positive]) were also calculated to assess the performance of diagnosing each disease.

We also used reductions in *k* to quantify the impact of changed sample conditions; specifically, Δ*k* quantifies the impact of the condition in classification performance.

### Removal of replicates in statistical analysis.

To avoid the statistical bias due to longitudinal sampling from the same host, we removed the replicated microbiomes in statistical analysis of both step I and step II. In step I, among replicates sampled from the same person in each cohort, only one sample was randomly selected and kept in calculating the results of the Wilcoxon rank-sum test for MNS. Likewise, during the cross-validation of step II, replicates from the same person in each cohort were also removed by random selection as in step I before calculating the Kappa coefficient/recall/precision for MSE and the machine learning-based approaches.

### Contamination simulation and test.

The reagent blank contamination OTUs were from the reagent blank microbiomes of the American Gut Project (Qiita ID 10317; 415 reagent blank samples in total; http://americangut.org/). The indoor environmental contamination OTUs were from the indoor environment microbiomes of the Qiita public database (11 studies; Table S3). All contamination source microbiomes were profiled the same way as the baseline samples and test samples. With a target test sample (the number of mapped reads is *m*) and a given contamination rate *r%*, a contamination source microbiome sample was randomly selected, and *m* × *r%* OTUs were randomly extracted from this sample and mixed into the OTU table of the target sample. The baseline databases of step I and the disease databases (training samples) of step II were kept as they were (i.e., with no contamination), while contaminations to Data Set Gut were simulated as described above to test the robustness of the MSE and the three model-based methods to contaminations.

10.1128/mSystems.00150-20.6 TABLE S3 Indoor microbiome used as sources of contamination. Download Table S3, DOCX file, 0.03 MB. Copyright © 2020 Su et al. 2020 Su et al. This content is distributed under the terms of the Creative Commons Attribution 4.0 International license.

10.1128/mSystems.00150-20.7 TABLE S4 Comparison of MSE and model approaches by CBH data sets. Download Table S4, DOCX file, 0.02 MB. Copyright © 2020 Su et al. 2020 Su et al. This content is distributed under the terms of the Creative Commons Attribution 4.0 International license.

### Evaluation of running speed.

To compare the speed of multiple classification between MSE and machine learning methods, we prepared a simulated data set with different numbers of gut microbiome samples (3,000, 6,000, 9,000, 12,000 or 15,000) as training data for model-based approaches (which also serve as the database for MSE) and another 100 samples as testing data (which also serve as the query for MSE). For model-based machine learning methods, the total running time includes both training time and testing time. For MSE, the total running time is for a complete two-step diagnosis with database construction and search (Fig. S2). All tests were performed on a single rack server node with Quad Intel Xeon E7-4820 CPUs, and multiple threads were enabled for applicable packages (MSE and XGBoost).

### Availability of data and materials.

MSE is developed and implemented in C/C++. It comes with a full automatic installer for cross-platform installation and setup in Linux/Mac OS X. The indexing and searching algorithm is optimized for parallel computing based on multiple CPUs using the OpenMP library. MSE accepts OTU tables for both database construction and search and thus is compatible with QIIME/QIIME 2, Parallel-META 3, and many other microbiome profiling tools. Both the source code and executive binary application packages are available at http://mse.ac.cn. The source code is also posted to the GitHub repository at http://github.com/qibebt-bioinfo/meta-storms. We have also developed a QIIME 2 plugin, which can be found at http://github.com/qibebt-bioinfo/q2-metastorms. In addition, all tests in this work were implemented as Linux shell scripts under Linux/Mac OS X to facilitate reproduction of the results. Scripts and test data sets are available at the download page of MSE (http://mse.ac.cn).

### Data availability.

The source data underlying Fig. 2A to D, 3A to H, 4A to D, 5A to F and Fig. S1, S2, and S3 are provided as Data Set S1. The baseline database samples and contamination samples, including sequence files and metadata, are available from Qiita (http://qiita.ucsd.edu) using the study IDs listed in Table S1 and Table S3. Data sets for the evaluation of search-based diagnosis, including sequence files and metadata, are available from the papers listed in Table S2. All other relevant data are available upon request.
