# Supplementary figures and images for: Multiple-Disease Detection and Classification across Cohorts via Microbiome Search
Source: mSystems. 2020 Mar 17;5(2):e00150-20. doi: 10.1128/mSystems.00150-20 (PMC7380586; doi:10.1128/mSystems.00150-20)

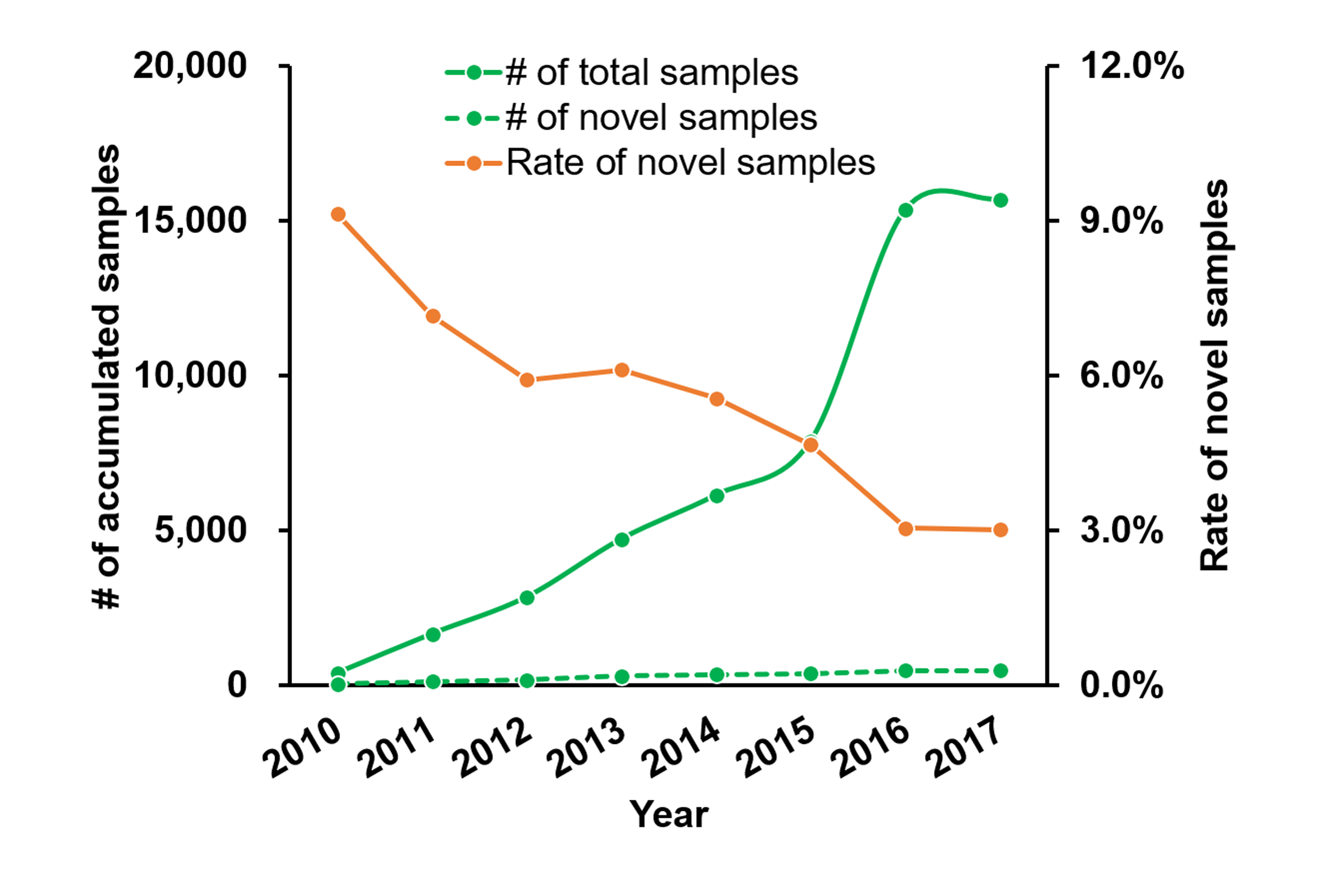

Supplement: FIG S1 [file mSystems.00150-20-sf001.tif]

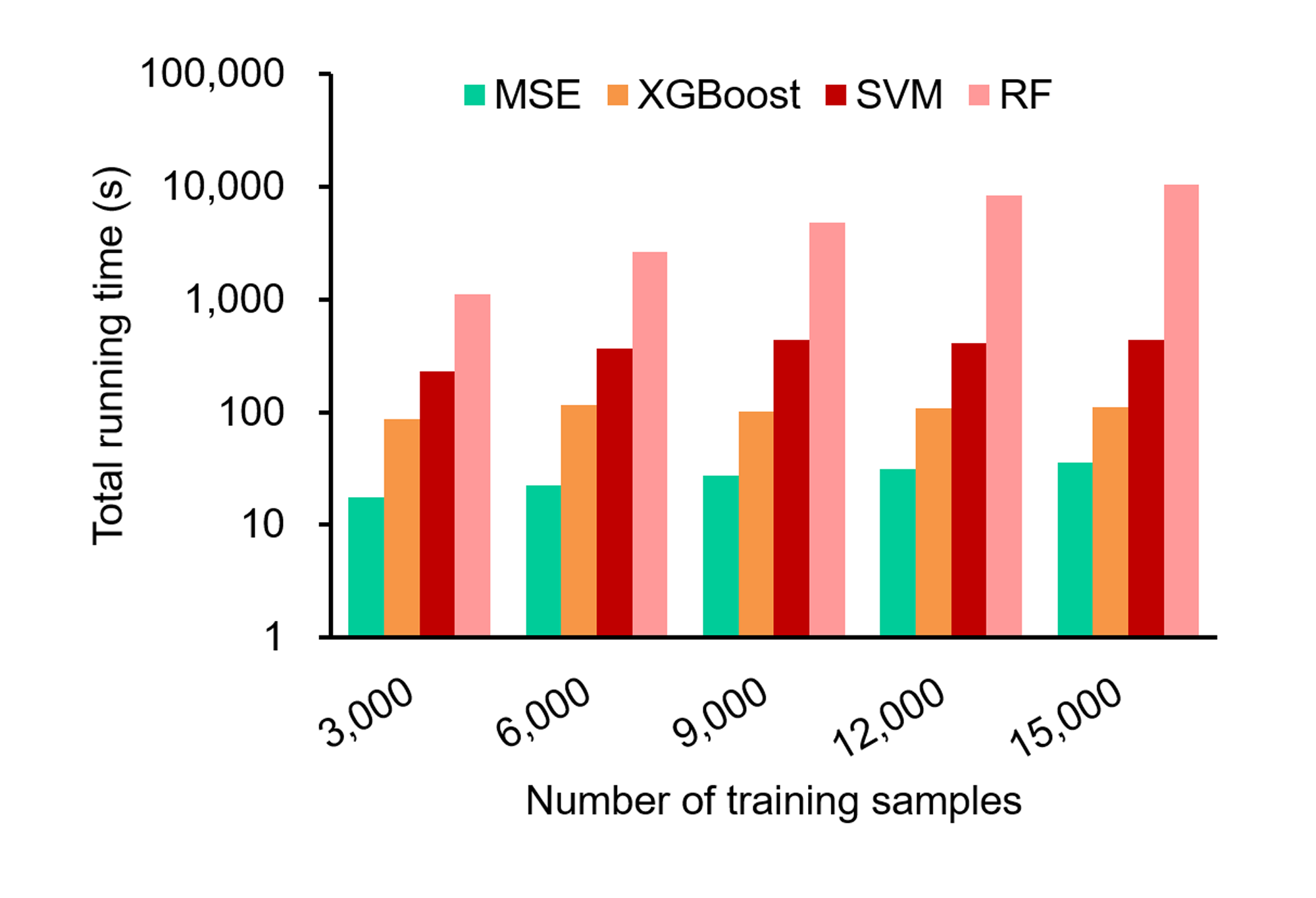

Supplement: FIG S2 [file mSystems.00150-20-sf002.tif]

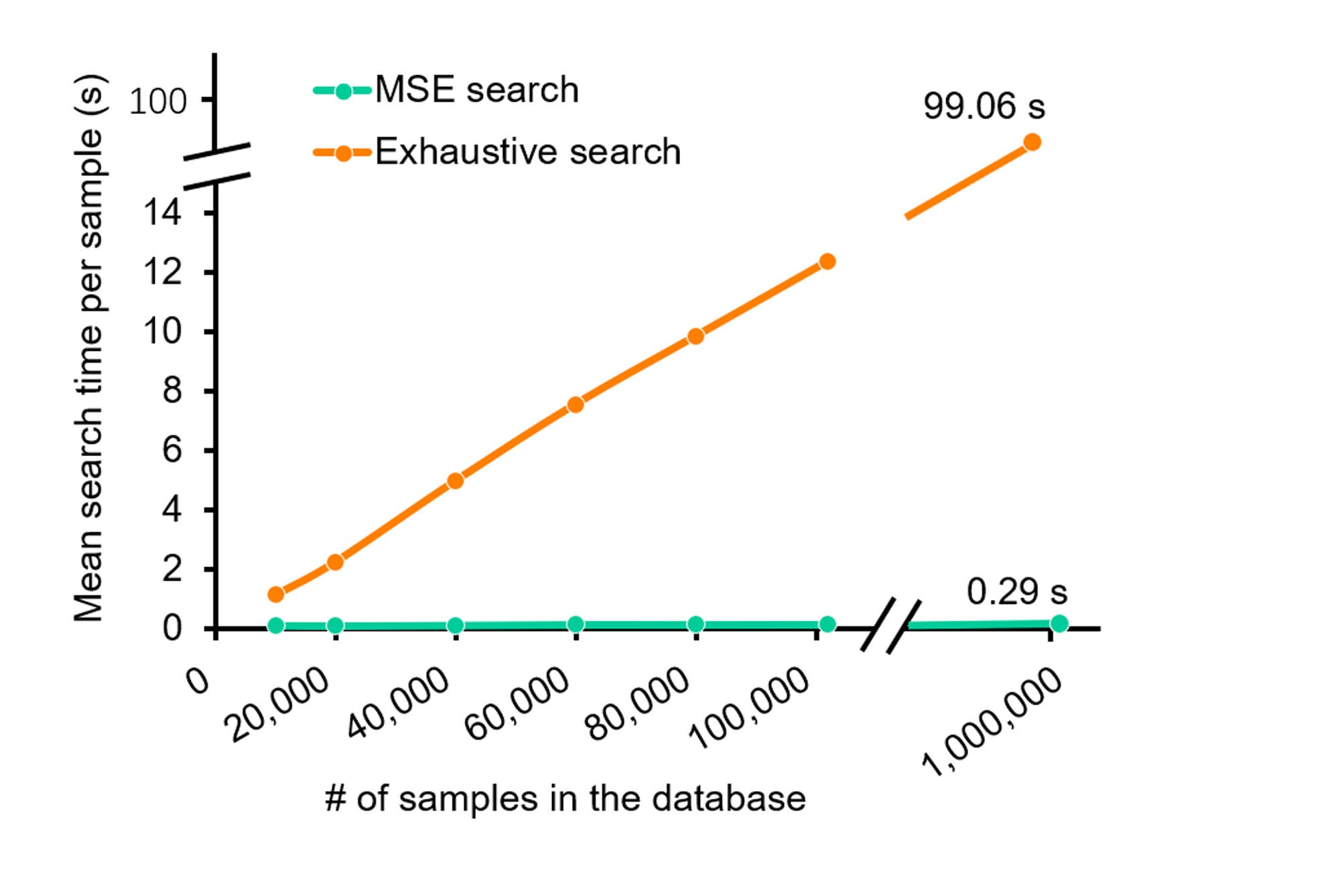

Supplement: FIG S3 [file mSystems.00150-20-sf003.tif]
