# Supplementary material for: Multiple-Disease Detection and Classification across Cohorts via Microbiome Search
Source: mSystems. 2020 Mar 17;5(2):e00150-20. doi: 10.1128/mSystems.00150-20 (PMC7380586; doi:10.1128/mSystems.00150-20)
Supplement: TABLE S1 [file mSystems.00150-20-st001.docx]

**Table S1. The fecal samples of baseline database for search-based diagnosis**

| Qiita ID | Project title | Num. of samples | Reference |
| --- | --- | --- | --- |
| 101 | Succession of microbial consortia in the developing infant gut microbiome | 60 | Koening, et al., PNAS, 2011 (1) |
| 349 | Human oral, gut, and plaque microbiota in patients with atherosclerosis | 15 | Koren, et al., PNAS, 2011 (2) |
| 353 | Effect of storage conditions on the assessment of bacterial community structure in soil and human-associated samples | 55 | Lauber, et al., FEMS Microbiol. Lett., 2010 (3) |
| 395 | Delivery mode effects on newborn microbiota | 7 | Dominguez-Bello, et al., PNAS, 2010 (4) |
| 449 | Bacterial community variation in human body habitats across space and time | 45 | NA |
| 458 | The effect of diet on the human gut microbiome: a metagenomic analysis in humanized gnotobiotic mice - human twins | 15 | Turnbaugh, et al., Sci. Transl. Med., 2009 (5) |
| 486 | Composition, variability, and temporal stability of the intestinal microbiota of the elderly | 100 | Claesson, et al., PNAS, 2011 (6) |
| 495 | Resistant starches types 2 and 4 have differential effects on the composition of the fecal microbiota in human subjects | 153 | NA |
| 550 | Moving pictures of the human microbiome | 467 | Caporaso, et al., Genome Biol., 2011 (7) |
| 737 | Gut microbiomes of Malawian twin pairs discordant for kwashiorkor - V2 | 971 | NA |
| 797 | Cohabiting family members share microbiota with one another and with their dogs | 168 | Song, et al., Elife, 2013 (8) |
| 850 | Human gut microbiome differentiation viewed across cultures, ages and families illumina | 528 | Yatsunenko, et al., Nature, 2012 (9) |
| 867 | Host remodeling of the gut microbiome and metabolic changes during pregnancy | 492 | Koren, et al., Cell, 2012 (10) |
| 959 | Widespread colonization of the lung by Tropheryma whipplei in HIV infection - CO samples | 11 | Lozupone, et al., Am. J. Respir. Crit. Care Med., 2013 (11) |
| 1010 | Linking Long-Term Dietary Patterns with Gut Microbial Enterotypes (cafe) | 95 | Wu, et al., Science, 2011 (12) |
| 1011 | Linking Long-Term Dietary Patterns with Gut Microbial Enterotypes (COMBO) | 99 | Wu, et al., Science, 2011 (12) |
| 1189 | Samples for the AGP from the PGP | 21 | NA |
| 1448 | Subsistence strategies in traditional societies distinguish gut microbiomes | 56 | Obregon-Tito, et al., Nat. Commun., 2015 (13) |
| 1481 | Ercolini whole grain feces | 88 | NA |
| 1561 | Characterization of the fecal microbiota using high-throughput sequencing reveals a stable microbial community during storage. | 50 | Carroll, et al., PLoS One, 2012 (14) |
| 1683 | Ultra-high-throughput microbial community analysis on the Illumina HiSeq and MiSeq platforms | 2 | Caporaso, et al., ISME J., 2012 (15) |
| 1684 | Ultra-high-throughput microbial community analysis on the Illumina HiSeq and MiSeq platforms (MiSeq) | 2 | Caporaso, et al., ISME J., 2012 (15) |
| 1774 | Puerto Rico and Plantanal | 122 | NA |
| 1927 | Structure, function and diversity of the healthy human microbiome (V13) | 197 | Human Microbiome Project (16) |
| 1928 | Structure, function and diversity of the healthy human microbiome (V35) | 360 | Human Microbiome Project (16) |
| 2010 | Partial restoration of the microbiota of cesarean-born infants via vaginal microbial transfer | 8 | Dominguez-Bello, et al., Nat. Med., 2016 (17) |
| 2014 | Human Genetics Shape the Gut Microbiome (MiSeq) | 1017 | Goodrich, et al., Cell, 2014 (18) |
| 2024 | The microbiota at multiple body sites during pregnancy in a rural Tanzanian population and the effects of Moringa supplemented probiotic yogurt. | 354 | NA |
| 2136 | Wischmeyer_ICU_microbiome | 31 | NA |
| 2202 | Host lifestyle affects human microbiota on daily timescales | 524 | David, et al., Genome Biol., 2014 (19) |
| 10052 | The microbiome of uncontacted Amerindians | 11 | Clemente, et al., Sci. Adv., 2015 (20) |
| 10057 | Changes in Microbial Ecology after Fecal Microbiota Transplantation for recurrent C. difficile Infection Affected by Underlying Inflammatory Bowel Disease | 105 | Khanna., et al., Microbiome, 2017 (21) |
| 10080 | The Fecal Microbial Community of Breast-fed Infants from Armenia and Georgia | 22 | NA |
| 10184 | Mayo Fecal Stability Study II | 962 | NA |
| 10218 | Improved Bacterial 16S rRNA Gene (V4 and V4-5) and Fungal Internal Transcribed Spacer Marker Gene Primers for Microbial Community Surveys | 5 | Walters, et al., mSystems, 2016 (22) |
| 10249 | Antibiotics, birth mode, and diet shape microbiome maturation during early life | 1216 | NA |
| 10257 | SIV fecal microbiome | 1 | Vujkovic-Cvijin, Cell Rep., 2015 (23) |
| 10293 | Impact of freeze-drying on milk oligosaccharide content and marker gene bacterial community profiles in infant fecal samples | 18 | NA |
| 10297 | Growth and Morbidity of Gambian Infants are Influenced by Maternal Milk Oligosaccharides and Infant Gut Microbiota | 5 | Davis, et al., Sci. Rep., 2017 (24) |
| 10300 | The Fecal Microbial Community of Breast-fed Infants from Georgia and Armenia | 1 | Lewis, et al., Sci. Rep., 2017 (25) |
| 10317 | American Gut Project | 5671 | American Gut Project (26) |
| 10342 | Gut microbiome diversity among Cheyenne and Arapaho individuals from western Oklahoma | 58 | Sankaranarayanan, et al., Curr. Biol., 2015 (27) |
| 10394 | Effects of preservation and storage conditions on the fecal microbiome | 887 | Song, et al., mSystems, 2016 (28) |
| 10483 | Role of Gut Microbiota in Pathophysiology of Parkinson's Disease | 6 | NA |
| 10512 | The pediatric intestinal mucosal microbiome remains altered after clinical resolution of inflammatory and ischemic disease | 6 | Wieck, et al., Surgery, 2016 (29) |
| 10530 | Mother’s Secretor Status Affects Development of Children’s Microbiota Composition and Function: A Pilot Study. | 37 | NA |
| 10532 | Microbiota Transfer Therapy alters gut ecosystem and improves gastrointestinal and autism symptoms: an open-label study | 241 | Kang, et al., Microbiome, 2017 (30) |
| 10564 | Baseline Fecal Samples of Hemapoetic Stem Cell Transplant Recipients and Donors | 59 | NA |
| 10567 | Parkinson’s disease and therapeutic modalities have distinct signatures on the gut microbiome | 208 | NA |
| 10589 | STAMPS 2016 Tutorial | 8 | NA |
| 10768 | Qiita Multidata Example | 13 | NA |
| 10778 | Faecal microbiota and Fat Free Mass Index in 2 to 3 year old well-nourished Australian children | 36 | Smith-Brown, Sci. Rep., 2016 (31) |
| 10988 | jtangApril2017CMI workshop | 15 | NA |

The original references were provided if the samples are published in journals. NA: not available.

**References**

1. Koenig JE, Spor A, Scalfone N, Fricker AD, Stombaugh J, Knight R, Angenent LT, Ley RE. 2011. Succession of microbial consortia in the developing infant gut microbiome. Proc Natl Acad Sci U S A 108 Suppl 1:4578-85.

2. Koren O, Spor A, Felin J, Fak F, Stombaugh J, Tremaroli V, Behre CJ, Knight R, Fagerberg B, Ley RE, Backhed F. 2011. Human oral, gut, and plaque microbiota in patients with atherosclerosis. Proc Natl Acad Sci U S A 108 Suppl 1:4592-8.

3. Lauber CL, Zhou N, Gordon JI, Knight R, Fierer N. 2010. Effect of storage conditions on the assessment of bacterial community structure in soil and human-associated samples. FEMS Microbiol Lett 307:80-6.

4. Dominguez-Bello MG, Costello EK, Contreras M, Magris M, Hidalgo G, Fierer N, Knight R. 2010. Delivery mode shapes the acquisition and structure of the initial microbiota across multiple body habitats in newborns. Proc Natl Acad Sci U S A 107:11971-5.

5. Turnbaugh PJ, Ridaura VK, Faith JJ, Rey FE, Knight R, Gordon JI. 2009. The effect of diet on the human gut microbiome: a metagenomic analysis in humanized gnotobiotic mice. Sci Transl Med 1:6ra14.

6. Claesson MJ, Cusack S, O'Sullivan O, Greene-Diniz R, de Weerd H, Flannery E, Marchesi JR, Falush D, Dinan T, Fitzgerald G, Stanton C, van Sinderen D, O'Connor M, Harnedy N, O'Connor K, Henry C, O'Mahony D, Fitzgerald AP, Shanahan F, Twomey C, Hill C, Ross RP, O'Toole PW. 2011. Composition, variability, and temporal stability of the intestinal microbiota of the elderly. Proc Natl Acad Sci U S A 108 Suppl 1:4586-91.

7. Caporaso JG, Lauber CL, Costello EK, Berg-Lyons D, Gonzalez A, Stombaugh J, Knights D, Gajer P, Ravel J, Fierer N, Gordon JI, Knight R. 2011. Moving pictures of the human microbiome. Genome Biol 12:R50.

8. Song SJ, Lauber C, Costello EK, Lozupone CA, Humphrey G, Berg-Lyons D, Caporaso JG, Knights D, Clemente JC, Nakielny S, Gordon JI, Fierer N, Knight R. 2013. Cohabiting family members share microbiota with one another and with their dogs. Elife 2:e00458.

9. Yatsunenko T, Rey FE, Manary MJ, Trehan I, Dominguez-Bello MG, Contreras M, Magris M, Hidalgo G, Baldassano RN, Anokhin AP, Heath AC, Warner B, Reeder J, Kuczynski J, Caporaso JG, Lozupone CA, Lauber C, Clemente JC, Knights D, Knight R, Gordon JI. 2012. Human gut microbiome viewed across age and geography. Nature 486:222-7.

10. Koren O, Goodrich JK, Cullender TC, Spor A, Laitinen K, Backhed HK, Gonzalez A, Werner JJ, Angenent LT, Knight R, Backhed F, Isolauri E, Salminen S, Ley RE. 2012. Host remodeling of the gut microbiome and metabolic changes during pregnancy. Cell 150:470-80.

11. Lozupone C, Cota-Gomez A, Palmer BE, Linderman DJ, Charlson ES, Sodergren E, Mitreva M, Abubucker S, Martin J, Yao G, Campbell TB, Flores SC, Ackerman G, Stombaugh J, Ursell L, Beck JM, Curtis JL, Young VB, Lynch SV, Huang L, Weinstock GM, Knox KS, Twigg H, Morris A, Ghedin E, Bushman FD, Collman RG, Knight R, Fontenot AP, Lung HIVMP. 2013. Widespread colonization of the lung by Tropheryma whipplei in HIV infection. Am J Respir Crit Care Med 187:1110-7.

12. Wu GD, Chen J, Hoffmann C, Bittinger K, Chen YY, Keilbaugh SA, Bewtra M, Knights D, Walters WA, Knight R, Sinha R, Gilroy E, Gupta K, Baldassano R, Nessel L, Li H, Bushman FD, Lewis JD. 2011. Linking long-term dietary patterns with gut microbial enterotypes. Science 334:105-8.

13. Obregon-Tito AJ, Tito RY, Metcalf J, Sankaranarayanan K, Clemente JC, Ursell LK, Zech Xu Z, Van Treuren W, Knight R, Gaffney PM, Spicer P, Lawson P, Marin-Reyes L, Trujillo-Villarroel O, Foster M, Guija-Poma E, Troncoso-Corzo L, Warinner C, Ozga AT, Lewis CM. 2015. Subsistence strategies in traditional societies distinguish gut microbiomes. Nat Commun 6:6505.

14. Carroll IM, Ringel-Kulka T, Siddle JP, Klaenhammer TR, Ringel Y. 2012. Characterization of the fecal microbiota using high-throughput sequencing reveals a stable microbial community during storage. PLoS One 7:e46953.

15. Caporaso JG, Lauber CL, Walters WA, Berg-Lyons D, Huntley J, Fierer N, Owens SM, Betley J, Fraser L, Bauer M, Gormley N, Gilbert JA, Smith G, Knight R. 2012. Ultra-high-throughput microbial community analysis on the Illumina HiSeq and MiSeq platforms. ISME J 6:1621-4.

16. Peterson J, Garges S, Giovanni M, McInnes P, Wang L, Schloss JA, Bonazzi V, McEwen JE, Wetterstrand KA, Deal C, Baker CC, Di Francesco V, Howcroft TK, Karp RW, Lunsford RD, Wellington CR, Belachew T, Wright M, Giblin C, David H, Mills M, Salomon R, Mullins C, Akolkar B, Begg L, Davis C, Grandison L, Humble M, Khalsa J, Little AR, Peavy H, Pontzer C, Portnoy M, Sayre MH, Starke-Reed P, Zakhari S, Read J, Watson B, Guyer M. 2009. The NIH Human Microbiome Project. Genome Res 19:2317-23.

17. Dominguez-Bello MG, De Jesus-Laboy KM, Shen N, Cox LM, Amir A, Gonzalez A, Bokulich NA, Song SJ, Hoashi M, Rivera-Vinas JI, Mendez K, Knight R, Clemente JC. 2016. Partial restoration of the microbiota of cesarean-born infants via vaginal microbial transfer. Nat Med 22:250-3.

18. Goodrich JK, Waters JL, Poole AC, Sutter JL, Koren O, Blekhman R, Beaumont M, Van Treuren W, Knight R, Bell JT, Spector TD, Clark AG, Ley RE. 2014. Human genetics shape the gut microbiome. Cell 159:789-99.

19. David LA, Materna AC, Friedman J, Campos-Baptista MI, Blackburn MC, Perrotta A, Erdman SE, Alm EJ. 2014. Host lifestyle affects human microbiota on daily timescales. Genome Biol 15:R89.

20. Clemente JC, Pehrsson EC, Blaser MJ, Sandhu K, Gao Z, Wang B, Magris M, Hidalgo G, Contreras M, Noya-Alarcon O, Lander O, McDonald J, Cox M, Walter J, Oh PL, Ruiz JF, Rodriguez S, Shen N, Song SJ, Metcalf J, Knight R, Dantas G, Dominguez-Bello MG. 2015. The microbiome of uncontacted Amerindians. Sci Adv 1.

21. Khanna S, Vazquez-Baeza Y, Gonzalez A, Weiss S, Schmidt B, Muniz-Pedrogo DA, Rainey JF, 3rd, Kammer P, Nelson H, Sadowsky M, Khoruts A, Farrugia SL, Knight R, Pardi DS, Kashyap PC. 2017. Changes in microbial ecology after fecal microbiota transplantation for recurrent C. difficile infection affected by underlying inflammatory bowel disease. Microbiome 5:55.

22. Walters W, Hyde ER, Berg-Lyons D, Ackermann G, Humphrey G, Parada A, Gilbert JA, Jansson JK, Caporaso JG, Fuhrman JA, Apprill A, Knight R. 2016. Improved Bacterial 16S rRNA Gene (V4 and V4-5) and Fungal Internal Transcribed Spacer Marker Gene Primers for Microbial Community Surveys. mSystems 1.

23. Vujkovic-Cvijin I, Swainson LA, Chu SN, Ortiz AM, Santee CA, Petriello A, Dunham RM, Fadrosh DW, Lin DL, Faruqi AA, Huang Y, Apetrei C, Pandrea I, Hecht FM, Pilcher CD, Klatt NR, Brenchley JM, Lynch SV, McCune JM. 2015. Gut-Resident Lactobacillus Abundance Associates with IDO1 Inhibition and Th17 Dynamics in SIV-Infected Macaques. Cell Rep 13:1589-97.

24. Davis JC, Lewis ZT, Krishnan S, Bernstein RM, Moore SE, Prentice AM, Mills DA, Lebrilla CB, Zivkovic AM. 2017. Growth and Morbidity of Gambian Infants are Influenced by Maternal Milk Oligosaccharides and Infant Gut Microbiota. Sci Rep 7:40466.

25. Lewis ZT, Sidamonidze K, Tsaturyan V, Tsereteli D, Khachidze N, Pepoyan A, Zhgenti E, Tevzadze L, Manvelyan A, Balayan M, Imnadze P, Torok T, Lemay DG, Mills DA. 2017. The Fecal Microbial Community of Breast-fed Infants from Armenia and Georgia. Sci Rep 7:40932.

26. McDonald D, Hyde E, Debelius JW, Morton JT, Gonzalez A, Ackermann G, Aksenov AA, Behsaz B, Brennan C, Chen Y, DeRight Goldasich L, Dorrestein PC, Dunn RR, Fahimipour AK, Gaffney J, Gilbert JA, Gogul G, Green JL, Hugenholtz P, Humphrey G, Huttenhower C, Jackson MA, Janssen S, Jeste DV, Jiang L, Kelley ST, Knights D, Kosciolek T, Ladau J, Leach J, Marotz C, Meleshko D, Melnik AV, Metcalf JL, Mohimani H, Montassier E, Navas-Molina J, Nguyen TT, Peddada S, Pevzner P, Pollard KS, Rahnavard G, Robbins-Pianka A, Sangwan N, Shorenstein J, Smarr L, Song SJ, Spector T, Swafford AD, Thackray VG, et al. 2018. American Gut: an Open Platform for Citizen Science Microbiome Research. mSystems 3.

27. Sankaranarayanan K, Ozga AT, Warinner C, Tito RY, Obregon-Tito AJ, Xu J, Gaffney PM, Jervis LL, Cox D, Stephens L, Foster M, Tallbull G, Spicer P, Lewis CM. 2015. Gut Microbiome Diversity among Cheyenne and Arapaho Individuals from Western Oklahoma. Curr Biol 25:3161-9.

28. Song SJ, Amir A, Metcalf JL, Amato KR, Xu ZZ, Humphrey G, Knight R. 2016. Preservation Methods Differ in Fecal Microbiome Stability, Affecting Suitability for Field Studies. mSystems 1.

29. Wieck MM, Debelius JW, Spurrier RG, Trecartin A, Knight R, Grikscheit TC. 2016. The pediatric intestinal mucosal microbiome remains altered after clinical resolution of inflammatory and ischemic disease. Surgery 160:350-8.

30. Kang DW, Adams JB, Gregory AC, Borody T, Chittick L, Fasano A, Khoruts A, Geis E, Maldonado J, McDonough-Means S, Pollard EL, Roux S, Sadowsky MJ, Lipson KS, Sullivan MB, Caporaso JG, Krajmalnik-Brown R. 2017. Microbiota Transfer Therapy alters gut ecosystem and improves gastrointestinal and autism symptoms: an open-label study. Microbiome 5:10.

31. Smith-Brown P, Morrison M, Krause L, Davies PS. 2016. Dairy and plant based food intakes are associated with altered faecal microbiota in 2 to 3 year old Australian children. Sci Rep 6:32385.
