# Supplementary material for: Multiple-Disease Detection and Classification across Cohorts via Microbiome Search
Source: mSystems. 2020 Mar 17;5(2):e00150-20. doi: 10.1128/mSystems.00150-20 (PMC7380586; doi:10.1128/mSystems.00150-20)
Supplement: TABLE S2 [file mSystems.00150-20-st002.docx]

**Table S2. Dataset Gut for evaluation of search-based diagnosis**

| Status | 16S Region | Platform | Num. of samples | Reference |
| --- | --- | --- | --- | --- |
| IBD and Control | V4 | Illumina HiSeq | 617 | Halfvarson et al., Nat. Microbio., 2017 (1) |
| IBD and Control | V4 | Illumina MiSeq | 708 | Gevers et al., Cell Host Microbe, 2014 (2) |
| HIV and Control | V4 | Illumina MiSeq | 51 | Lozupone etl al., Cell Host Microbe, 2013 (3) |
| HIV and Control | V3-V4 | Illumina MiSeq | 359 | Noguera-Julian et al, EBioMedicine, 2016 (4) |
| HIV and Control | V3-V5 | Roche 454 | 36 | Dinh et al., J. Infect. Dis., 2015 (5) |
| CRC | V4 | Illumina MiSeq and Roche 454 | 120 | Baxter et al., Genome Med., 2016 (6) |
| EDD | V3-V5 | Roche 454 | 222 | Singh et al., Microbiome, 2015 (7) |
| Control | V4 | Illumina HiSeq and MiSeq | 900 | American Gut Project (8) |
| Control | V1-V3 and V3-V5 | Roche 454 | 100 | Human Microbiome Project (9) |

**References**

1. Halfvarson J, Brislawn CJ, Lamendella R, Vazquez-Baeza Y, Walters WA, Bramer LM, D'Amato M, Bonfiglio F, McDonald D, Gonzalez A, McClure EE, Dunklebarger MF, Knight R, Jansson JK. 2017. Dynamics of the human gut microbiome in inflammatory bowel disease. Nat Microbiol 2:17004.

2. Gevers D, Kugathasan S, Denson LA, Vazquez-Baeza Y, Van Treuren W, Ren B, Schwager E, Knights D, Song SJ, Yassour M, Morgan XC, Kostic AD, Luo C, Gonzalez A, McDonald D, Haberman Y, Walters T, Baker S, Rosh J, Stephens M, Heyman M, Markowitz J, Baldassano R, Griffiths A, Sylvester F, Mack D, Kim S, Crandall W, Hyams J, Huttenhower C, Knight R, Xavier RJ. 2014. The treatment-naive microbiome in new-onset Crohn's disease. Cell Host Microbe 15:382-392.

3. Lozupone CA, Li M, Campbell TB, Flores SC, Linderman D, Gebert MJ, Knight R, Fontenot AP, Palmer BE. 2013. Alterations in the gut microbiota associated with HIV-1 infection. Cell Host Microbe 14:329-39.

4. Noguera-Julian M, Rocafort M, Guillen Y, Rivera J, Casadella M, Nowak P, Hildebrand F, Zeller G, Parera M, Bellido R, Rodriguez C, Carrillo J, Mothe B, Coll J, Bravo I, Estany C, Herrero C, Saz J, Sirera G, Torrela A, Navarro J, Crespo M, Brander C, Negredo E, Blanco J, Guarner F, Calle ML, Bork P, Sonnerborg A, Clotet B, Paredes R. 2016. Gut Microbiota Linked to Sexual Preference and HIV Infection. EBioMedicine 5:135-46.

5. Dinh DM, Volpe GE, Duffalo C, Bhalchandra S, Tai AK, Kane AV, Wanke CA, Ward HD. 2015. Intestinal microbiota, microbial translocation, and systemic inflammation in chronic HIV infection. J Infect Dis 211:19-27.

6. Baxter NT, Ruffin MTt, Rogers MA, Schloss PD. 2016. Microbiota-based model improves the sensitivity of fecal immunochemical test for detecting colonic lesions. Genome Med 8:37.

7. Singh P, Teal TK, Marsh TL, Tiedje JM, Mosci R, Jernigan K, Zell A, Newton DW, Salimnia H, Lephart P, Sundin D, Khalife W, Britton RA, Rudrik JT, Manning SD. 2015. Intestinal microbial communities associated with acute enteric infections and disease recovery. Microbiome 3:45.

8. McDonald D, Hyde E, Debelius JW, Morton JT, Gonzalez A, Ackermann G, Aksenov AA, Behsaz B, Brennan C, Chen Y, DeRight Goldasich L, Dorrestein PC, Dunn RR, Fahimipour AK, Gaffney J, Gilbert JA, Gogul G, Green JL, Hugenholtz P, Humphrey G, Huttenhower C, Jackson MA, Janssen S, Jeste DV, Jiang L, Kelley ST, Knights D, Kosciolek T, Ladau J, Leach J, Marotz C, Meleshko D, Melnik AV, Metcalf JL, Mohimani H, Montassier E, Navas-Molina J, Nguyen TT, Peddada S, Pevzner P, Pollard KS, Rahnavard G, Robbins-Pianka A, Sangwan N, Shorenstein J, Smarr L, Song SJ, Spector T, Swafford AD, Thackray VG, et al. 2018. American Gut: an Open Platform for Citizen Science Microbiome Research. mSystems 3.

9. Peterson J, Garges S, Giovanni M, McInnes P, Wang L, Schloss JA, Bonazzi V, McEwen JE, Wetterstrand KA, Deal C, Baker CC, Di Francesco V, Howcroft TK, Karp RW, Lunsford RD, Wellington CR, Belachew T, Wright M, Giblin C, David H, Mills M, Salomon R, Mullins C, Akolkar B, Begg L, Davis C, Grandison L, Humble M, Khalsa J, Little AR, Peavy H, Pontzer C, Portnoy M, Sayre MH, Starke-Reed P, Zakhari S, Read J, Watson B, Guyer M. 2009. The NIH Human Microbiome Project. Genome Res 19:2317-23.
