# Supplementary material for: Multiple-Disease Detection and Classification across Cohorts via Microbiome Search
Source: mSystems. 2020 Mar 17;5(2):e00150-20. doi: 10.1128/mSystems.00150-20 (PMC7380586; doi:10.1128/mSystems.00150-20)
Supplement: TABLE S3 [file mSystems.00150-20-st003.docx]

**Table S3. Indoor microbiome used as sources of contamination**

| Qiita ID | Project title | Num. of samples | Reference |
| --- | --- | --- | --- |
| 10172 | Colonization and Succession of Hospital-Associated Bacteria | 4394 | Lax et al., Sci. Transl. Med., 2017 (1) |
| 2192 | Longitudinal analysis of microbial interaction between humans and the indoor environment | 670 | Lax et al., Science, 2014 (2) |
| 10502 | Impact of water heater temperature and water use frequency on the building plumbing microbiome | 427 | Ji et al., ISME J., 2017 (3) |
| 10105 | Mapping microbial ecosystems and spoilage-gene flow in breweries highlights patterns of contamination and resistance | 400 | Bokulich et al., Elife, 2015 (4) |
| 1742 | Sloan Toronto House Project | 373 | NA |
| 10481 | Trade-offs between microbiome diversity and productivity in a stratified microbial mat | 268 | NA |
| 2019 | Microbial biogeography of wine grapes is conditioned by cultivar, vintage, and climate | 262 | Bokulich et al., PNAS, 2014 (5) |
| 1772 | Home Life: Factors Structuring the Bacterial Diversity Found within and between Homes HiSeq | 190 | Dunn et al., PLOS ONE, 2013 (6) |
| 1802 | Home Life: Factors Structuring the Bacterial Diversity Found within and between Homes MiSeq | 179 | Dunn et al., PLOS ONE, 2013 (6) |
| 1798 | Surface microbes in the neonatal intensive care unit: changes with routine cleaning and over time | 138 | Bokulich et al., J. Clin. Microbiol., 2013 (7) |

The original references were provided if the samples are published in journals. NA: not available.

**References**

1. Lax S, Sangwan N, Smith D, Larsen P, Handley KM, Richardson M, Guyton K, Krezalek M, Shogan BD, Defazio J, Flemming I, Shakhsheer B, Weber S, Landon E, Garcia-Houchins S, Siegel J, Alverdy J, Knight R, Stephens B, Gilbert JA. 2017. Bacterial colonization and succession in a newly opened hospital. Sci Transl Med 9.

2. Lax S, Smith DP, Hampton-Marcell J, Owens SM, Handley KM, Scott NM, Gibbons SM, Larsen P, Shogan BD, Weiss S, Metcalf JL, Ursell LK, Vazquez-Baeza Y, Van Treuren W, Hasan NA, Gibson MK, Colwell R, Dantas G, Knight R, Gilbert JA. 2014. Longitudinal analysis of microbial interaction between humans and the indoor environment. Science 345:1048-52.

3. Ji P, Rhoads WJ, Edwards MA, Pruden A. 2017. Impact of water heater temperature setting and water use frequency on the building plumbing microbiome. ISME J 11:1318-1330.

4. Bokulich NA, Bergsveinson J, Ziola B, Mills DA. 2015. Mapping microbial ecosystems and spoilage-gene flow in breweries highlights patterns of contamination and resistance. Elife 4.

5. Bokulich NA, Thorngate JH, Richardson PM, Mills DA. 2014. Microbial biogeography of wine grapes is conditioned by cultivar, vintage, and climate. Proc Natl Acad Sci U S A 111:E139-48.

6. Dunn RR, Fierer N, Henley JB, Leff JW, Menninger HL. 2013. Home Life: Factors Structuring the Bacterial Diversity Found within and between Homes. PLOS ONE 8:e64133.

7. Bokulich NA, Mills DA, Underwood MA. 2013. Surface microbes in the neonatal intensive care unit: changes with routine cleaning and over time. J Clin Microbiol 51:2617-24.
