# Supplementary material for: Multiple-Disease Detection and Classification across Cohorts via Microbiome Search
Source: mSystems. 2020 Mar 17;5(2):e00150-20. doi: 10.1128/mSystems.00150-20 (PMC7380586; doi:10.1128/mSystems.00150-20)
Supplement: TABLE S4 [file mSystems.00150-20-st004.docx]

**Table S4. Comparison of MSE and model approaches by CBH datasets**

| Method | PCC | RCI |
| --- | --- | --- |
| SVM | 0.92 | 0.77 |
| RF | 0.93 | 0.78 |
| KNN | 0.71 | 0.17 |
| MSE | 0.92 | 0.78 |

The CBH dataset is produced by Statnikov *et al Microbiome* 2013 (1). It contains *n*=552 human microbiome samples for six body sites. In Statnikov et al, 2013, the authors used the proportion of correct classifications (PCC) and relative classifier information (RCI) to benchmark the performance of machine learning methods in multiple classification of body sites.

**References**

1. Statnikov A, Henaff M, Narendra V, Konganti K, Li Z, Yang L, Pei Z, Blaser MJ, Aliferis CF, Alekseyenko AV. 2013. A comprehensive evaluation of multicategory classification methods for microbiomic data. Microbiome 1:11.
